# Intersectionality of gender, race/skin color and dental loss in adults: cross-sectional study with data from the 2019 Brazilian National Health Survey

**DOI:** 10.1590/S2237-96222025v34e20240524.en

**Published:** 2025-08-04

**Authors:** Bianca de Oliveira Farias, Larissa Fortunato Araújo, Nayranne Hivina Carvalho Tavares, Bárbara Bruna Rodrigues de Oliveira, Mayra Solange Lopes de Vasconcelos, Soraia Pinheiro Machado

**Affiliations:** 1 Universidade Estadual do Ceará, Centro de Ciências da Saúde, Programa de Pós-Graduação em Saúde Coletiva, Fortaleza, CE, Brazil; 2 Universidade Federal do Ceará, Centro de Ciências da Saúde, Programa de Pós-Graduação em Saúde Pública, Fortaleza, CE, Brazil; 3 Universidade Estadual do Ceará, Centro de Ciências da Saúde, Programa de Pós-Graduação em Nutrição e Saúde, Fortaleza, Ceará, Brazil

**Keywords:** Intersectional Framework, Intersectionality, Dental Extraction, Social Inequity, Cross-Sectional Studies, Marco interseccional, Interseccionalidad, Extracción dental, Desigualdad social, Estudios transversales

## Abstract

**Objective:**

To evaluate the association between intersectionality of gender, race/skin color and dental loss of 13 or more teeth among Brazilian adults.

**Methods:**

Cross-sectional study using data from the 2019 Brazilian National Health Survey. The response variable was dental loss of 13 or more teeth, the exposure was the intersectionality of gender, race/skin color, and the association was analyzed using logistic regression. Odds ratio (OR) and 95% confidence intervals (95%CI) were calculated. Sequential adjustments were made for age, education and macroregion of Brazil.

**Results:**

Among the 73,813 adults, higher odds of dental loss were observed in white women (OR 1.38 [95%CI 1.21; 1.56]) and Black women (OR 1.70 [95%CI 1.53; 1.89]) compared to white men, even after full adjustment. Among Black men, no significant differences were observed compared to white men regarding dental loss.

**Conclusion:**

Dental loss is associated with the intersectionality of gender, race/skin color. White and Black women appear to be more susceptible to dental loss. Social mechanisms such as racism and sexism may be intrinsically linked to the observed patterns of dental loss, highlighting the need to include actions on these mechanisms in existing national oral health policies, prioritizing disadvantaged segments.

## Resumen

**Objetivo:**

Evaluar la asociación entre la interseccionalidad de género, raza/color de piel y la pérdida de 13 o más dientes entre adultos brasileños.

**Métodos:**

Estudio transversal realizado con datos de la Encuesta Nacional de Salud 2019. La variable respuesta fue la pérdida de 13 o más dientes, la exposición fue la interseccionalidad de género, raza/color de piel y la asociación se analizó mediante regresión logística. Se estimaron los *odds ratios* (OR) y los intervalos de confianza del 95% (IC95%). Se realizaron ajustes secuenciales por edad, educación y macrorregión de Brasil.

**Resultados:**

Entre los 73.813 adultos, se observaron mayores probabilidades de pérdida de dientes en mujeres blancas (OR 1,38; [IC95% 1,21; 1,56]) y mujeres negras (OR 1,70; [IC95% 1,53; 1,89]) en comparación con los hombres blancos, incluso después del ajuste completo. Entre los hombres negros, no se observaron diferencias significativas en comparación con los hombres blancos con respecto a la pérdida de dientes.

**Conclusión:**

La pérdida de dientes se asocia con la interseccionalidad de género, raza/color de piel. Las mujeres blancas y negras parecen ser más susceptibles a la ocurrencia de pérdida de dientes. Los mecanismos sociales como el racismo y el sexismo pueden estar intrínsecamente vinculados a los patrones observados de pérdida de dientes, lo que destaca la necesidad de incluir acciones sobre los mecanismos en las políticas nacionales de salud bucal existentes, priorizando los segmentos desfavorecidos.

**Ethical aspects**

This research respected ethical principles, having obtained the following approval data::

Research Ethics Committee: Comissão Nacional de Ética em Pesquisa

Opinion number: 3.529.376

Approval date: 8/23/2019

Certificate of Submission for Ethical Appraisal: 11713319.7.0000.0008

Informed Consent Form: Obtained from all participants prior to collection of samples.

## Introduction

Dental loss, especially significant loss that impacts functionality, represents a public health problem in Brazil (1), and expresses social and health inequities and inequalities experienced by part of the population (2). Data from the previous edition of the 2013 Brazilian National Health Survey (PNS) indicate that of those aged 18 or over, 11.0% had lost all their teeth, while the loss of 13 or more teeth was reported by 23.0% of adults and the elderly. Both types of losses were more frequent in women, the elderly, people with low levels of education, and those living in rural areas (1). 

Historically disadvantaged groups have higher prevalence of untreated cavities, dental pain, and infections, as well as greater difficulty in accessing dental services, reflecting signs of social exclusion that manifest themselves in worse health conditions (3). The difficulty in timely access to dental services, as well as conservative treatment, partly explains why dental extraction is considered the most viable option, especially among people with low income (4).

Some physiological conditions help to understand the greater tooth loss among women, as well as the greater occurrence of cavities and systemic diseases due to hormonal conditions (1,5). However, social issues expressed by sexism can have a greater impact on this process. Less access to the job market, lower income, greater economic dependence, and the precarious working conditions to which women are exposed can affect health (6-7), contributing to worse oral health conditions and greater dental loss, compared to men (8). 

It is also important to recognize racism as a central component in the formation of inequalities in health, including those related to oral health, particularly in Brazil, which is a country marked by miscegenation and social inequalities (9), which can affect access, quality of access and use of health services (10). Several studies report a higher incidence of dental loss among mixed-race and Black Brazilians, when compared to whites (1,10-131), especially among Black women (12).

In this context, the theory of intersectionality between gender, race/skin color explores the complexity and interdependence of various social identities, which makes it possible to understand how the social constructions that preceded the lives of individuals generate various levels of inclusion or exclusion in health (13-14). The association of race/skin color and gender helps to highlight the lived realities that elucidate inequities (15), especially in oral health. Although some research examine how isolated social markers can impact oral health (8,16-17), the intersectionality of gender, race/skin color and their implications for oral health is still little explored (12), especially among Black women, who are so marginalized. 

The main innovation of this work is the application of the intersectional perspective to identify how structural inequalities impact dental loss, a gap still little explored in Brazilian literature. Considering intersectionality and how certain population groups, especially Black women, have worse oral health conditions, which can affect dental loss, it is important that actions and policies are sensitive to the vulnerabilities of this group. Research on the point just made is relevant to discuss strategies on how to reduce barriers to access to dental care and more specialized treatments, in order to overcome structural inequalities that impact oral health care, in addition to supporting the development of specific strategies to reduce disparities in access to dental care and providing valuable directions for the creation of more effective and equitable oral health programs. Thus, the aim of our study was to evaluate the association between intersectionality of gender, race/skin color and the loss of 13 or more teeth among Brazilian adults participating in a population-based study.

## Methods

### Study design

Cross-sectional, population-based study using data from PNS 2019, a Brazilian population survey developed by the Ministry of Health and the Oswaldo Cruz Foundation, in partnership with the Brazilian Institute of Geography and Statistics (IBGE). The PNS aimed to produce data on the health status and lifestyle of the Brazilian population, based on information from rural and urban households in the country.

### Context

The PNS information was collected through a computer-assisted interview with the head of the household or an eligible resident of each selected household who was over 14 years old.

The probabilistic sampling procedure was used to select the participating holseholds and was prepared from a master sample composed of 8,036 primary sampling units which satisfied the criterion that, in Federation Units (UF) with a greater number of primary sampling units, the defined number of households would be smaller, with 12 households for interview for each of them. In turn, in the UFs with a smaller number of primary sampling units, the defined number of households would be greater, with 18 households for interview for each of them. Finally, in the other UFs that did not meet these criteria, the number allocated was 15 households for interview per primary sampling unit. All information about the methodological procedures can be found in the specific publication (18). The 2019 PNS had 94,114 participating households and obtained a response rate of 96.5%. 

### Participants

To meet the inclusion criteria for this study, adults aged 18 to 64 years were considered, with the aim of standardizing the exposure time, since adolescents have not lived enough to have the study’s dental loss of more than 13 teeth and have better oral health compared to adults, and elderly people have other factors that lead to dental loss. Furthermore, those who had sociodemographic information and information on dental loss were included. Those who self-reported as Indigenous and as Indian/reddish/yellow skin color were excluded, as recommended by the IBGE, as this corresponds to a small number of observations with a high coefficient of variation (18). Thus, the study analysis was composed of 73,813 participants. 

### Variables

The response variable was the loss of 13 or more teeth, investigated based on the following questions from module U on oral health: “Thinking about your bottom teeth, have you lost any teeth?” and “Thinking about your upper teeth, have you lost any teeth?” Answers ranged from 1 (No), 2 (Yes, I lost teeth) and 3 (I lost all my upper/lower teeth). For people who reported having lost at least one tooth, both in the upper and lower jaw, the total number of teeth lost was recorded.

For analysis purposes, a combination of both questions was considered, which categorized tooth loss as <13 teeth and ≥13 teeth. This categorization was based on the number of teeth from which the dentition is considered dysfunctional (19). 

As an exposure variable, the intersectionality of gender, race/skin color was considered, composed of the combination of the independent variable of self-reported race/skin color (dichotomized into white and black – brown and black). Regarding the sex variable, the individual’s birth sex was collected; however, the biological sex was used as proxy for gender (male and female). Four categories of race/skin color and gender intersectionality were considered: White man (reference category), Black man, White woman, and Black woman.

For the assessment of sociodemographic aspects, the following were considered as covariates for adjustment: age (18-24, 25-34, 35-44, 45-54, and 55-64), current education (complete higher education or more, complete high school, complete elementary education, incomplete elementary education or no education), and macroregion of Brazil (Southeast, South, Midwest, Northeast and North). 

### Statistical methods

The study population was described using relative frequencies and their respective 95% confidence intervals (95%CI). Dental loss was analyzed according to the intersectionality categories of gender, race/skin color, in addition to other study variables.

The association between the intersectionality of gender, race/skin color, and the loss of 13 or more teeth was analyzed using logistic regression, obtaining odds ratios (OR) and their respective 95%CI (Model 0). Subsequently, sequential adjustment was made for age, current education, and macro-region of Brazil (Model 1). The data were analyzed using Stata software version 15.0, using the survey module for complex samples. 

## Results

 Black women (29.9% [95%CI 29.3; 30.5]), people aged between 35 and 44 years (23.8% [95%CI 23.2; 24.3]), with complete high school education (36.5% [95%CI 35.8; 37.2]) and residents in the Southeast region (42.5% [95%CI 41.5; 43.5]) were proportionally higher in the study population. The loss of 13 or more teeth was observed in women (14.3% [95%CI 13.7; 15.0]), brown people (13.1% [95%CI 12.5; 13.6]) compared to white men (11.4% [95%CI 10.7; 12.0]), those with incomplete elementary education or less (42.7% [95%CI 41.7; 43.7]) and among those residing in the Northeast region (15.3% [95%CI 14.7; 16.0). Also, from the age of 35, dental loss increases with age, being greater between the ages of 55 and 64 (43.2% [95%CI 41.8; 44.7]) (Table 1). When investigating the description of dental loss according to the intersectionality categories of gender, race/skin color, we noted that white women (12.9% [95%CI 11.8; 13.9]) and black women (15.5% [95%CI 14.6; 16.5]) presented higher prevalences of loss of 13 or more teeth compared to white men (9.7% [95%CI 8.9; 10.4]) (Table 2).

**Table 2:** Prevalence (%) and 95% confidence intervals (95%CI) of loss of 13 or more teeth by intersectionality categories of gender, race/skin color, Brazilian National Health Survey. Brazil, 2019

Study variables	White man % (IC95%)	Black man % (IC95%)	White woman % (IC95%)	Black woman % (IC95%)
**Tooth Loss ≥ 13 teeth**				
No	90.3 (89.5; 91.0)	89.8 (89.1; 90.5)	87.1 (86.0; 88.1)	84.5 (83.7; 85.3)
Yes	9.7 (8.9; 10.4)	10.2 (9.4; 10.8)	12.9 (11.8; 13.9)	15.5 (14.6; 16.5)

**Table 1:** Prevalence (%), absolute frequencies (n) and 95% confidence intervals (95%CI) of population characteristics due to the loss of 13 or more teeth, Brazilian National Health Survey. Brazil, 2019 (n=73,813)

Study variables	Total population		Tooth loss, <13 teeth		Tooth loss, >13 teeth	
	n (%)^a^	95%CI^b^	n (%)^a^	95%CI^b^	n (%)^a^	95%CI^b^
Gender						
Male	35,209 (47.7)	47.0; 48.4	31,723 (90.1)	89.5;90.6	3,486 (9.9)	9.4; 10.4
Female	38,604 (52.3)	51.6; 52.9	33,083 (85.7)	85.0;86.3	5,521 (14.3)	13.7; 15.0
**Race/skin color**						
White	31,149 (42.2)	41.4; 42.9	27,598 (88.6)	87.9;89.3	3,551 (11.4)	10.7; 12.0
Brown	34,028 (46.1)	45.3; 46.8	29,570 (86.9)	86.4;87.5	4,458 (13.1)	12.5; 13.6
Black	8,636 (11.7)	11.3; 12.2	7,582 (87.8)	86.5;89.1	1,054 (12.2)	10.9; 13.5
**Age** (years)						
18-24	12,032 (16.3)	15.7; 16.9	12,008 (99.8)	99.6:99.9	24 (0.2)	0.1; 1.1
25-34	15,722 (21.3)	20.7; 21.8	15,581 (99.1)	98.9;99.3	141 (0.9)	0.7; 1.1
35-44	17,567 (23.8)	23.2; 24.3	16,776 (95.5)	95.0;96.0	790 (4.5)	4.0; 5.0
45-54	15,501 (21.0)	20.5; 21.5	12,463 (80.4)	79.3;81.4	3,038 (19.6)	18.6; 20.7
55-64	12,991 (17.6)	17.2; 18.1	7,379 (56.8)	55.3;58.2	5,612 (43.2)	41.8; 44.7
Education						
Completed higher education or more	11,589 (15.7)	15.0; 16.3	10,940 (94.4)	93.8;95.0	649 (5.6)	5.0; 6.2
Completed high school	26,942 (36.5)	35.8; 37.2	24,894 (92.4)	91.9;92.9	2,048 (7.6)	7.1; 8.1
Completed elementary education	13,950 (18.9)	18.4; 19.5	12,248 (87.8)	86.7;88.7	1,702 (12.2)	11.2; 13.3
Incomplete elementary education or no education	21,332 (28.9)	28.2; 29.6	12,223 (57.3)	56.3;58.3	9,109 (42.7)	41.7; 43.7
**Macroregion of Brazil**						
Southeast	31,371 (42.5)	41.5; 43.5	28,140 (89.7)	88.9;90.5	3,231 (10.3)	9.5; 11.1
South	10,703 (14.5)	14.0; 14.9	9,290 (86.8)	85.9;87.7	1,413 (13.2)	12.3; 14.1
Midwest	5,757 (7.8)	7.5; 8.2	5,118 (88.9)	87.8;89.8	639 (11.1)	10.2; 12.2
Northeast	19,782 (26.8)	26.2; 27.5	16,755 (84.7)	84.0;85.3	3,027 (15.3)	14.7; 16.0
North	6.201(8.4)	8.0; 8.6	5,475 (88.3)	87.4;89.1	726 (11.7)	10.9; 12.5

^a^Relative and absolute frequencies

Table 3 describes the associations between intersectionality of gender, race/skin color and the loss of 13 or more teeth. We observed that white women (OR 1.38 [95%CI 1.21; 1.56]) and black women (OR 1.70 [95%CI 1.53; 1.89]) were more likely to have lost 13 or more teeth compared to white men (reference category) in the raw model (Model 0), with a greater magnitude of association for black women. After adjusting for age, education, and region of the country (Model 1), we did not see significant changes in the magnitudes of the associations, only a negative confounding effect. Black men did not differ from white men in terms of the loss of 13 or more teeth in either model. 

**Table 3:** Odds ratio (OR) and 95% confidence interval (95%CI) between intersectionality of gender, race/skin color and loss of 13 or more teeth, Brazilian National Health Survey. Brazil, 2019

	Model 0^a^ OR (95%CI)	Model 1^b^ OR (95%CI)
**Gender race intersectionality**		
White man	Reference	Reference
Black man	1.05 (0.92; 1.19)	0.92 (0.78; 1.07)
White woman	1.38 (1.21; 1.56)	1.55 (1.33; 1.80)
Black woman	1.70 (1.53; 1.89)	1.84 (1.62; 2.09)

^a^Model 0: raw; ^b^Model 1: adjusted for age, current education and macroregion of Brazil

## Discussion

In this study, we observed that white and Black women had greater chances of dental loss compared to white men, with greater magnitudes among Black women (black and brown). White and Black men did not differ in terms of the loss of 13 or more teeth.

A limitation that should be considered when analyzing our results is the fact that information about the loss of 13 or more teeth was self-reported, which may lead to information bias. However, some studies prove the validity of information on dental loss for the assessment of oral health, when reported by research participants themselves in Brazil (20) and the United States (21). In the PNS, these biases are minimized, as there is a standardized protocol for collecting, measuring, and interpreting information, standardized questionnaires, and training of interviewers for the instrument application. Another limitation is that the PNS only considered the sex of individuals, and we used this variable as a proxy for gender, which makes it difficult to identify social patterns related to gender, which go beyond the differences of the biological dichotomy. This is because by treating sex as a binary category, it is assumed that individuals’ gender identity is aligned with their birth sex.

It should be noted that, as this is a recent household survey, with a large population-based sample, this study offers a valuable opportunity to analyze inequalities in oral health, considering the intersectionality between gender and race/skin color in Brazil, a developing country characterized by deep social inequities. In agreement with the present study, the 2010 Brazilian National Oral Health Survey, conducted with 22,843 individuals, demonstrated that dental loss was more prevalent in women, older people, Black people, with lower education and income (2,17). Regarding education, in agreement with the present study, dental loss is proportionally higher in individuals with fewer years of education. This can be explained by the fact that education is strongly associated with the health status of the population, since the knowledge acquired allows for the adoption of healthier lifestyles. There is also a clear association of income and access to health services (3,22).

Although dental loss tends to increase with age (23), women, compared to men, have higher rates of tooth decay from adolescence onwards (24), suggesting that women begin to experience dental loss earlier than men due to tooth cavities. Despite this, the biological factors used to explain these differences cannot, by themselves, justify why health conditions affect individuals differently (25).

Although they have a greater concern about the aesthetics of their smile, take greater care with oral hygiene, and seek dental services more frequently than men, women have a higher prevalence of dental loss (7.24) and demonstrate a more negative self-perception of oral health (25). Furthermore, they have a higher prevalence of systemic diseases and are affected by hormonal conditions that can influence their oral health and, consequently, affect dental loss (8). Another influencing factor is eating habits. Brazilian women consume cakes, cookies, sweets, milk, and dairy products more frequently (26), which are fermentable carbohydrates with a direct positive relationship to the etiology of caries and periodontal disease, the main causes of dental loss (27).

Sexism explains how society is organized from the perspective of domination of one sex over the other, with greater privileges for men (28). Women generally face barriers in accessing the job market, have lower income and less education, greater economic dependence and more precarious working conditions, which negatively impacts their general health status (9), in addition to being directly related to worse oral health and, consequently, a higher incidence of dental loss (10).

We must consider that dental loss does not occur the same way among women, as factors such as race/skin color also influence this. Furthermore, racial disparities affect women and men differently. These disparities are caused by complex mechanisms that involve the intersection between gender and race/skin color, important markers of social position. Such social markers are reflected in different health indicators, both at the individual and contextual levels, revealing how oppression or privileges manifest themselves in diverse ways among population groups (13). 

Research conducted in São Paulo with 17,560 people reveals that black and brown individuals have more precarious oral conditions, with higher rates of cavities and dental loss (above 15 teeth), compared to those with white skin, reinforcing how certain racial groups are more vulnerable to socioeconomic differences (8).This can be understood by the way in which race/skin color establishes a significant social hierarchy, as it is at the beginning of the chain of effects resulting from socioeconomic differences (3). Furthermore, black people are more likely to have lower income and lower education (29), and a lower chance of black women not having access to oral health services in primary care, compared to white women (30), as well as less understanding about care and good oral hygiene habits (16) was also observed. Thus, it is clear that race/skin color is associated with socioeconomic status and greater social vulnerability in the context of oral health, resulting in less access to dental services and a greater need for accumulated treatment (1), as well as a greater number of missing teeth (31).

Despite evidence that black and brown people tend to have greater tooth loss compared to white people (1,10,12), this was not found among the men in our study, since no difference was observed between white and black men, indicating how oral health is more strongly impacted in women. Data from the Brazilian National Oral Health Survey of 2010 shows that, compared to men, women presented greater dental loss (25), and the same was found in our study, both for white and black women, although a greater magnitude of the association was observed in black women in relation to white women. A survey with 38,573 Black women in the United States showed that 17.0% of them rated the health of their teeth and gums as fair or poor, and 25.0% indicated they were missing at least one tooth (12). Black women are more likely to receive inappropriate treatment, putting them in a position of greater vulnerability, compromising their right to health and access to quality services (12,32). Thus, it is clear that the intersection of gender and race/skin color determines social disparities, hierarchizing access to health services. Despite sexism, racism determines the conditions of access for Black women, as they suffer more from poor access, while white women are prioritized for good access (33).

Lower socioeconomic level, prejudice and discrimination, in addition to the poorer quality of access to health services endured by this group, ends up making conservative care less accessible, since specialized dental treatments are still difficult to access in the Brazilian Unified Health System, which makes extraction the most viable option, especially for people with lower income, as they are more dependent on the public health service (29,33).

It should also be considered that black women (brown and black) suffer from low levels of education, gender-based violence (13), have jobs in less healthy environments, higher prevalence of excess weight, sedentary lifestyle (34), metabolic syndrome (35), and also tend to consume more foods rich in fat. Furthermore, brown women have lower consumption of fruits and vegetables and a higher prevalence of passive smoking in their homes (36). This affects the health of this group in general and places them in a more vulnerable condition regarding their oral health and dental loss.

This study found out that Black women (black and brown), with less education and living in the Northeast region of the country had a higher prevalence of loss of 13 or more teeth. A study carried out with 37,262 Brazilians from the National Program for Improving Access and Quality of Primary Care showed that black women living in the Northeast and North regions with low levels of education had worse access to preventive health services, as a result of mechanisms such as structural racism (30). In general, residents of the North and Northeast regions present greater losses than residents of the South and Southeast regions, reflecting the difference in coverage of tooth loss prevention, in addition to the lower use of and access to oral health services in poorer regions (37). Given the above, dental loss can be understood as a result not only of oral diseases, but also of the process of social, economic, and cultural exclusion throughout life. 

Furthermore, the repercussions that oral health problems can cause, in addition to the loss of the physiological function linked to chewing, are also configured as a social element of human interaction with the world and stigma in society, such as the impact on physical appearance and self-esteem, difficulties in getting a job and socializing, in addition to the reduction in economic productivity. This, in turn, can aggravate social, psychological, and financial issues, even leading to nutritional deficiencies, creating a vicious cycle that tends to worsen overall health (38).

Our study highlighted how sexism and racism directly impact dental loss in Brazil. By analyzing social relationships between men (white and black) and women (white and black), this study reveals how the intersections between gender and race/skin color can intensify inequalities in oral health, leading to disparities in access to and quality of dental care. Identifying the groups most vulnerable to dental loss provides a basis for directing financial resources and more precise public policies, allowing oral health actions to be better adapted to the specific needs of each group.

This knowledge enables the creation of more effective interventions aimed at improving the health and quality of life of the most affected populations, such as Black women, who historically face additional barriers in accessing dental care. Furthermore, the findings of this study can reinforce and improve public policies already in force, such as the National Oral Health Policy, the National Policy for Comprehensive Health of the Black Population, the National Policy for Comprehensive Health Care for Women, and the National Policy Plan for Women. By incorporating an intersectional perspective of gender and race/skin color, these policies can advance in the fight against inequalities, especially regarding Black women, ensuring fairer and more equitable access to oral health care.

## Data Availability

The PNS database and analysis codes used in the research are available at https://www.ibge.gov.br/estatisticas/ sociais/saude/9160-pesquisa-nacional-de-saude.html (37), while the methodological aspects, sampling plan and data analysis are available at https://www.scielo.br/j/ress/a/RdbtmCHjJGt8xDW6bV3Y6JB/ (18).
