# Supplementary material for: Intersectionality of gender, race/skin color and dental loss in adults: cross-sectional study with data from the 2019 Brazilian National Health Survey
Source: Epidemiol Serv Saude. 2025 Aug 4;34:e20240524. doi: 10.1590/S2237-96222025v34e20240524.en (PMC12334234; doi:10.1590/S2237-96222025v34e20240524.en)
Supplement: Translation

# Translation: Interseccionalidade gênero, raça/cor da pele e perda dentária em adultos: estudo transversal com dados da Pesquisa Nacional de Saúde 2019

**DOI:** 10.1590/S2237-96222025v34e20240524.pt

**Authors:** Bianca de Oliveira Farias, Larissa Fortunato Araújo, Nayranne Hivina Carvalho Tavares, Bárbara Bruna Rodrigues de Oliveira, Mayra Solange Lopes de Vasconcelos, Soraia Pinheiro Machado

**Affiliations:** 1 Universidade Estadual do Ceará, Centro de Ciências da Saúde, Programa de Pós-Graduação em Saúde Coletiva, Fortaleza, CE, Brasil; 2 Universidade Federal do Ceará, Centro de Ciências da Saúde, Programa de Pós-Graduação em Saúde Pública, Fortaleza, CE, Brasil; 3 Universidade Estadual do Ceará, Centro de Ciências da Saúde, Programa de Pós-Graduação em Nutrição e Saúde, Fortaleza, Ceará, Brasil

**Keywords:** Enquadramento Interseccional, Interseccionalidade, Extração dentária, Iniquidade Social, Estudos Transversais.

## Abstract

**Objetivo:**

Avaliar a associação entre interseccionalidade gênero, raça/cor da pele e a perda dentária de 13 dentes ou mais entre adultos brasileiros.

**Métodos:**

Estudo transversal realizado com dados da Pesquisa Nacional de Saúde 2019. A variável resposta foi perda dentária de 13 dentes ou mais, a exposição foi a interseccionalidade gênero, raça/cor da pele, e a associação foi analisada por meio de regressão logística. Estimaram-se as razões de chances (*odds ratio*, OR) e intervalos de confiança de 95% (IC95%). Ajustamentos sequenciais foram realizados por idade, escolaridade e macrorregião do Brasil.

**Resultados:**

Entre os 73.813 adultos, maiores chances de perda dentária foram observadas em mulheres brancas (OR 1,38 [IC95% 1,21; 1,56]) e negras (OR 1,70 [IC95% 1,53; 1,89]) se comparadas aos homens brancos, mesmo após completo ajustamento. Entre homens negros, não se observaram diferenças significativas se comparados aos homens brancos quanto à perda dentária.

**Conclusão:**

A perda dentária está associada à interseccionalidade gênero, raça/cor da pele. Mulheres brancas e negras parecem estar mais suscetíveis à ocorrência de perda dentária. Mecanismos sociais como racismo e sexismo podem estar intrinsecamente ligados aos padrões de perda dentária observados, evidenciando a necessidade da inserção de ações sobres mecanismos nas políticas nacionais de saúde bucal já existentes, priorizando os segmentos desfavorecidos.

**Aspectos éticos**

A presente pesquisa respeitou os princípios éticos, obtendo os seguintes dados de aprovação: :

Comitê de ética em pesquisa: Comissão Nacional de Ética em Pesquisa

Número do parecer: 3.529.376

Data de aprovação: 23/8/2019

Certificado de apresentação de apreciação ética: 11713319.7.0000.0008

Registro do consentimento livre e esclarecido: Obtido de todos os participantes antes da coleta.

## Introdução

A perda de dentes, especialmente uma perda significativa que impacta a funcionalidade, representa um problema de saúde pública no Brasil (1), e expressa iniquidades e desigualdades sociais e de saúde experienciadas por parte da população (2). Dados da edição prévia da Pesquisa Nacional de Saúde (PNS), em 2013, apontam que, das pessoas com 18 anos ou mais de idade, 11,0% perderam todos os dentes, enquanto a perda de 13 ou mais dentes foi referida por 23,0% dos adultos e idosos. Ambas as perdas foram mais frequentes em mulheres, idosos, pessoas de baixa escolaridade, e que residem em área rural (1). 

Grupos historicamente desfavorecidos têm maiores prevalências de cáries não tratadas, dores e infecções dentárias, além de maior dificuldade no acesso aos serviços odontológicos, refletindo os sinais da exclusão social que se manifestam em piores condições de saúde (3). A dificuldade no acesso precoce ao serviço odontológico, bem como o tratamento conservador, explicam, em parte, por que a extração dentária é considerada a opção mais viável, especialmente entre pessoas com baixa renda (4).

Algumas condições fisiológicas ajudam a entender a maior perda dentária entre mulheres, como a maior ocorrência de cáries e doenças sistêmicas em decorrência das condições hormonais (1,5). Entretanto, questões sociais expressas pelo sexismo podem ter maior impacto neste processo. Menor acesso ao mercado de trabalho, menor renda, maior dependência econômica e as precárias condições de trabalho a que as mulheres são expostas podem afetar a saúde (6-7), contribuindo para piores condições de saúde bucal e maior perda dentária, em comparação aos homens (8). 

Também é importante reconhecer o racismo como um componente central na formação de desigualdades em saúde, inclusive relacionadas à saúde bucal, particularmente no Brasil, que é um país marcado por miscigenação e desigualdades sociais (9), as quais podem afetar o acesso, a qualidade do acesso e a utilização dos serviços de saúde (10). Diversos estudos reportam maior ocorrência de perda dentária entre pardos e pretos brasileiros, quando comparados aos brancos (1,10-131), sobretudo entre mulheres pretas (12).

Nesse contexto, a teoria da interseccionalidade gênero, raça/cor da pele explora a complexidade e a interdependência de várias identidades sociais, o que possibilita entender como as construções sociais que antecedem a vida dos indivíduos geram diferentes níveis de inclusão ou exclusão em saúde (13-14). Relacionar raça/cor da pele e gênero contribui para evidenciar as realidades vividas que elucidam as iniquidades (15), especialmente na saúde bucal. Embora as pesquisas analisem como os marcadores sociais isolados podem impactar a saúde bucal (8,16-17), a interseccionalidade gênero, raça/cor da pele e suas implicações na saúde bucal ainda é pouco explorada (12), principalmente entre as mulheres negras que são tão marginalizadas. 

A principal inovação deste trabalho está na aplicação da perspectiva interseccional para identificar como as desigualdades estruturais impactam a perda dentária, uma lacuna ainda pouco explorada na literatura brasileira. Considerando a interseccionalidade e como determinados grupos populacionais, sobretudo as mulheres negras, apresentam piores condições de saúde bucal, que podem afetar a perda dentária, é importante que ações e políticas sejam sensíveis às vulnerabilidades desse grupo. Pesquisas nessa perspectiva são relevantes para discutir estratégias de como diminuir as barreiras de acesso aos cuidados odontológicos e a tratamentos mais especializados, a fim de superar as desigualdades estruturais que impactam o cuidado em saúde bucal, além de subsidiar o desenvolvimento de estratégias específicas para reduzir as disparidades no acesso ao cuidado odontológico e fornecer direções valiosas para a criação de programas de saúde bucal mais eficazes e equitativos. Assim, o objetivo do nosso estudo foi avaliar a associação entre interseccionalidade gênero, raça/cor da pele e a perda dentária de 13 ou mais dentes entre adultos brasileiros participantes de um estudo de base populacional.

## Métodos

### Delineamento

Estudo transversal, de base populacional, utilizando dados da PNS 2019, um inquérito populacional brasileiro desenvolvido pelo Ministério da Saúde e pela Fundação Oswaldo Cruz, em parceria com o Instituto Brasileiro de Geografia e Estatística (IBGE). A PNS teve como objetivo produzir dados sobre a situação de saúde e estilo de vida da população brasileira, considerando informações de domicílios rurais e urbanos do país.

### Contexto

As informações da PNS foram levantadas por entrevista assistida por computador, com um morador responsável ou elegível de cada domicílio selecionado que tivesse idade superior a 14 anos.

Para a seleção dos domicílios participantes, utilizou-se o procedimento de amostragem probabilística, elaborado a partir de uma amostra mestra composta por 8.036 unidades primárias de amostragem que seguia o critério de que, em Unidades da Federação (UF) com maior quantitativo de unidade primárias de amostragem, a quantidade definida de domicílios seria menor, sendo 12 domicílios para entrevista para cada uma. Por seu turno, nas UF com menor quantitativo de unidade primárias de amostragem, a quantidade definida de domicílios seria maior, sendo 18 domicílios para entrevista por cada uma. Por fim, nas demais UF que não se enquadravam em tais critérios, a quantidade alocada foi de 15 domicílios para entrevista por unidade primária de amostragem. Todas as informações sobre os procedimentos metodológicos podem ser encontradas em publicação específica (18). A PNS 2019 contou com 94.114 domicílios participantes e obteve uma taxa de resposta de 96,5%. 

### Participantes

Como critérios de inclusão para o presente estudo, foram considerados adultos com idades de 18 a 64 anos, com o intuito de uniformizar o tempo de exposição, já que adolescentes têm um tempo menor de vida, melhor saúde bucal comparada aos adultos e menor perda dentária superior a 13 dentes, e idosos apresentam outros fatores que levam à perda dentária. Além disso, foram incluídos os que possuíam informações sociodemográficas e sobre perda dentária. Foram excluídos aqueles que autorrelataram raça/cor da pele indígena e amarela, conforme recomendação do IBGE, uma vez que corresponde a um pequeno número de observações com elevado coeficiente de variação (18). Assim, a análise do estudo foi composta por um quantitativo de 73.813 participantes. 

### Variáveis

A variável resposta foi a perda dentária de 13 ou mais dentes, investigada a partir das seguintes perguntas do módulo U sobre saúde bucal: “Lembrando-se dos seus dentes de baixo, o(a) sr(a) perdeu algum dente?” e “Lembrando-se dos seus dentes de cima, o(a) sr(a) perdeu algum dente?”. As respostas variaram entre 1 (Não), 2 (Sim, perdi) e 3 (Perdi todos os dentes de baixo/cima). Para as pessoas que relataram ter perdido ao menos um dente, tanto na arcada superior quanto na inferior da mandíbula, foi registrado o total de dentes perdidos.

Para fins de análise, foi considerada uma combinação de ambas as perguntas, que foram categorizadas em perda: <13 dentes e ≥13 dentes. Tal categorização foi baseada no número a partir do qual a dentição é considerada disfuncional (19). 

Como variável exposição, foi considerada a interseccionalidade gênero, raça/cor da pele, composta pela combinação da variável independente de raça/cor da pele autorreferida (dicotomizada em brancos e negros – pardos e pretos). Já em relação à variável sexo, foi coletado o sexo de nascimento do indivíduo; no entanto, utilizado o biológico como proxy de gênero (homem e mulher). Foram consideradas quatro categorias de interseccionalidade raça/cor da pele e gênero: homem branco (categoria de referência), homem negro, mulher branca e mulher negra.

Para levantamento dos aspectos sociodemográficos, foram consideradas como covariáveis para ajuste: idade (18-24, 25-34, 35-44, 45-54, e 55-64), escolaridade atual (superior completo ou mais, médio completo, fundamental completo, fundamental incompleto ou menos), e macrorregião do Brasil (Sudeste, Sul, Centro-Oeste, Nordeste e Norte). 

### Métodos estatísticos

A população do estudo foi descrita por meio de frequências relativas e seus respectivos intervalos de confiança de 95% (IC95%). A perda dentária foi analisada de acordo com as categorias de interseccionalidade gênero, raça/cor da pele, além das outras variáveis do estudo.

A associação entre a interseccionalidade gênero, raça/cor da pele, com a perda dentária de 13 ou mais dentes foi analisada por meio de regressão logística, obtendo-se razão de chances (*odds ratio*, OR) e seus respectivos IC95% (Modelo 0). Posteriormente, fez-se ajustamento sequencial por: idade, escolaridade atual e macrorregião do Brasil (Modelo 1). Os dados foram analisados por meio do software Stata versão 15.0, com uso de módulo survey para amostras complexas. 

## Resultados

Na população do estudo, mulheres negras (29,9% [IC95% 29,3; 30,5]), pessoas com idade entre 35 e 44 anos (23,8% [IC95% 23,2; 24,3]), com ensino médio completo (36,5% [IC95% 35,8; 37,2]) e residentes na região Sudeste (42,5% [IC95% 41,5; 43,5]) encontravam-se em maior proporção no estudo. A perda dentária de 13 ou mais dentes foi observada em mulheres (14,3% [IC95% 13,7; 15,0]), pardos (13,1% [IC95% 12,5; 13,6]) em relação aos brancos (11,4% [IC95% 10,7; 12,0]), naqueles com ensino fundamental incompleto ou menos (42,7% [IC95% 41,7; 43,7]) e entre os que residiam no Nordeste (15,3% [IC95% 14,7; 16,0). Também a partir de 35 anos, a perda dentária aumenta com a idade, sendo maior entre 55 e 64 anos (43,2% [IC95% 41,8; 44,7]) (Tabela 1). Ao investigar a descrição da perda dentária conforme as categorias de interseccionalidade gênero, raça/cor da pele, foi possível observar que mulheres brancas (12,9% [IC95% 11,8; 13,9]) e negras (15,5% [IC95% 14,6; 16,5]), apresentaram maiores prevalências de perda de 13 ou mais dentes comparados aos homens brancos (9,7% [IC95% 8,9; 10,4]) (Tabela 2).

**Tabela 1:** Prevalência (%), frequências absolutas (n) e intervalos de confiança de 95% (IC95%) das características da população por perda dentária de 13 ou mais dentes, Pesquisa Nacional de Saúde. Brasil, 2019 (n=73.813)

Variáveis do estudo	População total		Perda dentária, <13 dentes		Perda dentária, >13 dentes	
	n (%)^a^	IC95%^b^	n (%)^a^	IC95%^b^	n (%)^a^	IC95%^b^
Gênero						
Homem	35.209 (47,7)	47,0; 48,4	31.723 (90,1)	89,5;90,6	3.486 (9,9)	9,4; 10,4
Mulher	38.604 (52,3)	51,6; 52,9	33.083 (85,7)	85,0;86,3	5.521 (14,3)	13,7; 15,0
**Raça/cor da pele**						
Branca	31.149 (42,2)	41,4; 42,9	27.598 (88,6)	87,9;89,3	3.551 (11,4)	10,7; 12,0
Parda	34.028 (46,1)	45,3; 46,8	29.570 (86,9)	86,4;87,5	4.458 (13,1)	12,5; 13,6
Preta	8.636 (11,7)	11,3; 12,2	7.582 (87,8)	86,5;89,1	1.054 (12,2)	10,9; 13,5
**Idade** (anos)						
18-24	12.032 (16,3)	15,7; 16,9	12.008 (99,8)	99,6:99,9	24 (0,2)	0,1; 1,1
25-34	15.722 (21,3)	20,7; 21,8	15.581 (99,1)	98,9;99,3	141 (0,9)	0,7; 1,1
35-44	17.567 (23,8)	23,2; 24,3	16.776 (95,5)	95,0;96,0	790 (4,5)	4,0; 5,0
45-54	15.501 (21,0)	20,5; 21,5	12.463 (80,4)	79,3;81,4	3.038 (19,6)	18,6; 20,7
55-64	12.991 (17,6)	17,2; 18,1	7.379 (56,8)	55,3;58,2	5.612 (43,2)	41,8; 44,7
Escolaridade						
Superior completo ou mais	11.589 (15,7)	15,0; 16,3	10.940 (94,4)	93,8;95,0	649 (5,6)	5,0; 6,2
Médio completo	26.942 (36,5)	35,8; 37,2	24.894 (92,4)	91,9;92,9	2.048 (7,6)	7,1; 8,1
Fundamental completo	13.950 (18,9)	18,4; 19,5	12.248 (87,8)	86,7;88,7	1.702 (12,2)	11,2; 13,3
Fundamental incompleto ou menos	21.332 (28,9)	28,2; 29,6	12.223 (57,3)	56,3;58,3	9.109 (42,7)	41,7; 43,7
**Macroregião do Brasil**						
Sudeste	31.371 (42,5)	41,5; 43,5	28.140 (89,7)	88,9;90,5	3.231 (10,3)	9,5; 11,1
Sul	10.703 (14,5)	14,0; 14,9	9.290 (86,8)	85,9;87,7	1.413 (13,2)	12,3; 14,1
Centro-Oeste	5.757 (7,8)	7,5; 8,2	5.118 (88,9)	87,8;89,8	639 (11,1)	10,2; 12,2
Nordeste	19.782 (26,8)	26,2; 27,5	16.755 (84,7)	84,0;85,3	3.027 (15,3)	14,7; 16,0
Norte	6.201(8,4)	8,0; 8,6	5.475 (88,3)	87,4;89,1	726 (11,7)	10,9; 12,5

^a^Frequências relativas e absolutas.

**Tabela 2:** Prevalência (%) e intervalos de confiança de 95% (IC95%) da perda dentária de 13 ou mais dentes por categorias de interseccionalidade gênero, raça/cor da pele, Pesquisa Nacional de Saúde. Brasil, 2019

Variáveis do estudo	Homem branco % (IC95%)	Homem negro % (IC95%)	Mulher branca % (IC95%)	Mulher negra % (IC95%)
**Perda dentária** ≥**13 dentes**				
Não	90,3 (89,5; 91,0)	89,8 (89,1; 90,5)	87,1 (86,0; 88,1)	84,5 (83,7; 85,3)
Sim	9,7 (8,9; 10,4)	10,2 (9,4; 10,8)	12,9 (11,8; 13,9)	15,5 (14,6; 16,5)

Na tabela 3, estão descritas as associações entre interseccionalidade gênero, raça/cor da pele e perda dentária de 13 ou mais dentes. Observamos que mulheres, brancas (OR 1,38 [IC95% 1,21; 1,56]) e negras (OR 1,70 [IC95% 1,53; 1,89], apresentaram maiores chances de ter perda dentária de 13 ou mais dentes, se comparadas aos homens brancos (categoria de referência) no modelo bruto (Modelo 0), havendo maior magnitude da associação em mulheres negras. Após ajustamento por idade, escolaridade e região do país (Modelo 1), não vemos mudanças significativas nas magnitudes das associações, apenas um efeito de confusão negativo. Homens negros não diferiram dos homens brancos quanto à perda de 13 ou mais dentes em nenhum dos modelos. 

**Tabela 3:** Razão de chances (*odds ratio*, OR) e intervalo de confiança de 95% (IC95%) entre interseccionalidade gênero, raça/cor da pele e perda dentária de 13 ou mais dentes, Pesquisa Nacional de Saúde. Brasil, 2019

	Modelo 0^a^ OR (IC95%)	Modelo 1^b^ OR ( IC95%)
**Interseccionalidade gênero raça**		
Homem branco	Referência	Referência
Homem negro	1,05 (0,92; 1,19)	0,92 (0,78; 1,07)
Mulher branca	1,38 (1,21; 1,56)	1,55 (1,33; 1,80)
Mulher negra	1,70 (1,53; 1,89)	1,84 (1,62; 2,09)

^a^Modelo 0: bruto; ^b^Modelo 1: ajustado por idade, escolaridade atual e macrorregião do Brasil.

## Discussão

Observamos, neste estudo, que mulheres brancas e negras apresentaram maiores chances de perda dentária comparadas aos homens brancos, com maiores magnitudes entre as mulheres negras (pretas e pardas). Homens brancos e negros não diferiram quanto à perda dentária de 13 dentes ou mais.

Uma limitação que deve ser considerada na análise dos nossos resultados é o fato de que a informação sobre a perda dentária de 13 dentes ou mais ter foi autorrelatada, podendo haver viés de informação. Entretanto, há estudos que comprovam a validade de informações sobre perda dentária para avaliação da saúde bucal, quando referida pelos próprios participantes de pesquisas no Brasil (20) e nos Estados Unidos (21). Na PNS, esses vieses são minimizados, pois há um protocolo padronizado de coleta, medição e interpretação de informações, questionários padronizados, além do treinamento dos entrevistadores para aplicação do instrumento. Outra limitação é que a PNS considerou apenas o sexo dos indivíduos, e utilizamos essa variável como um proxy de gênero, o que dificulta a identificação de padrões sociais relacionados ao gênero, que vão além das diferenças da dicotomia biológica. Isso ocorre porque, ao tratar o sexo como uma categoria binária, pressupõe-se que a identidade de gênero dos indivíduos esteja alinhada com o sexo registrado no nascimento.

Ressalta-se que, por se tratar de uma pesquisa domiciliar recente, com grande amostra, de base populacional, este estudo oferece uma valiosa oportunidade para analisar as desigualdades em saúde bucal, considerando a interseccionalidade entre gênero e raça/cor da pele, no Brasil, um país em desenvolvimento, caracterizado por profundas iniquidades sociais. Concordando com o presente estudo, a Pesquisa Nacional de Saúde Bucal de 2010, feita com 22.843 indivíduos, demonstrou que a perda de dentes foi mais prevalente em mulheres, mais idosas, negras, com menor escolaridade e renda (2,17). Quanto à escolaridade, concordando com o presente estudo, proporções de perda dentária são maiores em indivíduos com menos anos de estudo. Isso pode ser explicado pelo fato de a escolaridade estar fortemente associada ao estado de saúde da população, uma vez que o conhecimento adquirido permite adotar estilos de vida mais saudáveis, além de haver uma clara conexão com a renda e o acesso aos serviços de saúde (3,22).

Embora a perda de dentes tenda a ser maior com o avançar da idade (23), as mulheres, comparadas aos homens, possuem índices mais elevados de cárie dentária desde a adolescência (24), o que sugere que mulheres começam a ter perda dentária mais cedo do que homens em virtude de cárie dentária. Apesar disso, os fatores biológicos utilizados para explicar essas diferenças não conseguem, por si sós, justificar por que as condições de saúde afetam os indivíduos de maneira distinta (25).

Embora possuam uma maior preocupação acerca da estética de seu sorriso, mais cuidado com a higiene oral, e busquem mais frequentemente os serviços odontológicos do que os homens, as mulheres apresentam maiores prevalências de perda dentária (7,24) e demonstram uma autopercepção de saúde bucal mais negativa (25). Além disso, apresentam maiores prevalências de doenças sistêmicas e são acometidas por condições hormonais que podem influenciar em sua saúde bucal e, consequentemente, afetar a perda dentária (8). Outro fator que influencia são os hábitos alimentares. Mulheres brasileiras consomem com maior frequência bolos, biscoitos, doces, leite e derivados (26), que são carboidratos fermentáveis com relação positiva direta para etiologia da cárie e doença periodontal, principais causas de perda dentária (27).

O sexismo explica como a sociedade se organiza sob a perspectiva de dominação de um sexo sobre o outro, com maiores privilégios para os homens (28). Mulheres geralmente enfrentam barreiras no acesso ao mercado de trabalho, têm menor renda e menor escolaridade, maior dependência econômica e condições de trabalho mais precárias, o que impacta negativamente o seu estado de saúde geral (9), além de se relacionar diretamente a pior saúde bucal e, consequentemente, maior incidência de perda dentária (10)

Devemos considerar que a perda dentária não ocorre de maneira uniforme entre mulheres, já que fatores como a raça/cor da pele também influenciam. Além disso, as disparidades raciais afetam mulheres e homens de formas diferentes. Essas disparidades são causadas por mecanismos complexos que envolvem a intersecção entre gênero e raça/cor da pele, importantes marcadores de posição social. Tais marcadores sociais se refletem em diferentes indicadores de saúde, tanto em nível individual quanto contextual, revelando como a opressão ou os privilégios se manifestam de maneiras distintas entre grupos populacionais (13). 

Pesquisa conduzida em São Paulo, envolvendo 17.560 pessoas, revela que indivíduos pretos e pardos apresentam condições bucais mais precárias, com índices mais altos de cáries e perda de dentes (acima de 15 unidades), em comparação com aqueles de pele branca, reforçando como determinados grupos raciais são mais vulneráveis às diferenças socioeconômicas (8).Isso pode ser compreendido pela maneira como a raça/cor da pele estabelece uma hierarquia social significativa, por estar no início da cadeia de efeitos decorrentes de diferenças socioeconômicas (3). Ademais, pessoas negras estão mais propensas a possuir menor renda e menor escolaridade (29), tendo sido observado ainda uma menor chance de mulheres negras não terem acesso a serviços de saúde bucal na atenção básica, se comparadas às brancas (30), e menor entendimento acerca de cuidados e bons hábitos de higiene bucal (16). Dessa forma, fica claro que a raça/cor da pele está associada à posição socioeconômica e à maior vulnerabilidade social no contexto da saúde bucal, resultando no menor acesso a serviços odontológicos e na maior necessidade de tratamento acumulado (1), bem como em maior número de dentes perdidos (31).

Apesar das evidências de que pessoas pretas e pardas tendem a apresentar maior perda dentária em comparação aos brancos (1,10,12), isso não foi encontrado entre os homens do nosso estudo, uma vez que não se observou diferença entre homens brancos e negros, indicando o quanto a saúde bucal é mais fortemente impactada nas mulheres. Dados da Pesquisa Brasileira de Saúde Bucal de 2010 mostram que, em comparação aos homens, mulheres apresentaram maior perda dentária (25), e o mesmo foi encontrado no nosso estudo, tanto para mulheres brancas quanto negras, embora maior magnitude da associação tenha sido observada na mulher negra em relação à mulher branca. Em uma pesquisa com 38.573 mulheres pretas nos Estados Unidos, 17,0% avaliaram a saúde de seus dentes e gengivas como regular ou ruim e 25,0% indicaram ter perdido pelo menos um dente (12). Mulheres negras são mais propensas a receber tratamentos inadequados, colocando-as em uma posição de maior vulnerabilidade, comprometendo seu direito à saúde e ao acesso a serviços de qualidade (12,32). Assim, fica evidente que a intersecção de gênero e raça/cor da pele determina as disparidades sociais, hierarquizando o acesso aos serviços de saúde. Apesar do sexismo, o racismo determina as condições de acesso das mulheres negras, pois sofrem mais com o acesso ruim, enquanto as mulheres brancas são prioridade no acesso bom (33).

O menor nível socioeconômico, o preconceito e a discriminação, além da pior qualidade do acesso aos serviços de saúde sofrida por esse grupo, acaba tornando o atendimento conservador menos acessível, uma vez que tratamentos odontológicos especializados ainda são de difícil acesso no Sistema Único de Saúde, o que faz com que a extração seja a opção mais viável, especialmente em pessoas com menor renda, por serem mais dependentes do serviço público de saúde (29,33).

Deve-se considerar também que mulheres negras (pardas e pretas) sofrem com a baixa escolaridade, violência de gênero (13), têm trabalhos em ambientes menos saudáveis, maiores prevalências de excesso de peso, sedentarismo (34), síndrome metabólica (35), bem como consomem mais alimentos ricos em gordura. Além disso, mulheres pardas apresentam menor consumo de frutas e hortaliças e maior prevalência de serem fumantes passivas em seu domicílio (36). Isso afeta a saúde desse grupo de forma geral e o coloca numa condição de maior vulnerabilidade quanto à sua saúde bucal e à perda dentária.

Neste estudo, mulheres negras (pretas e pardas), com menor escolaridade e residentes na região Nordeste do país apresentaram maior prevalência de perda dentária de 13 ou mais dentes. Estudo realizado com 37.262 brasileiros do Programa Nacional de Melhoria do Acesso e da Qualidade da Atenção Básica evidenciou que mulheres negras residentes da região Nordeste e Norte com baixa escolaridade tinham pior acesso a serviços preventivos de saúde, em decorrência de mecanismos como o racismo estrutural (30). De maneira geral, residentes das regiões Norte e Nordeste apresentam maiores perdas do que os residentes das regiões Sul e Sudeste, refletindo a diferença da cobertura de preventivas de perdas dentárias, além do menor uso e acesso aos serviços de saúde bucal em regiões mais pobres (37). Diante do exposto, evidencia-se que as perdas dentárias podem ser entendidas como resultado não apenas das doenças bucais, mas também do processo de exclusão social, econômica e cultural no percurso de vida. 

Ademais, as repercussões que problemas de saúde bucal podem acarretar, além da perda da função fisiológica ligada à mastigação, também se configuram como um elemento social de interação do ser humano com o mundo e do estigma na sociedade, como o impacto na aparência física e na autoestima, dificuldades em conseguir emprego e se socializar, além da redução da produtividade econômica. Isso pode, por sua vez, agravar questões sociais, psicológicas e financeiras, chegando até a provocar deficiências nutricionais, criando um ciclo vicioso que tende a piorar o estado geral de saúde (38).

Nosso estudo evidenciou como o sexismo e o racismo impactam diretamente a perda dentária no Brasil. Ao analisar as relações sociais entre homens (brancos e negros) e mulheres (brancas e negras), este estudo revela como as intersecções entre gênero e raça/cor da pele podem intensificar as desigualdades em saúde bucal, levando a disparidades no acesso e na qualidade do cuidado odontológico. A identificação dos grupos mais vulneráveis à perda dentária oferece uma base para o direcionamento de recursos financeiros e políticas públicas mais precisas, permitindo que as ações de saúde bucal sejam mais bem adaptadas às necessidades específicas de cada grupo.

Esse conhecimento possibilita a criação de intervenções mais eficazes, voltadas para a melhoria da saúde e qualidade de vida das populações mais afetadas, como as mulheres negras, que historicamente enfrentam barreiras adicionais no acesso ao cuidado odontológico. Além disso, os achados deste estudo podem reforçar e aprimorar políticas públicas já em vigor, como a Política Nacional de Saúde Bucal, a Política Nacional de Saúde Integral da População Negra, a Política Nacional de Assistência Integral à Saúde da Mulher e o Plano Nacional de Políticas para Mulheres. Essas políticas, ao incorporarem uma perspectiva interseccional de gênero e raça/cor da pele, podem avançar no combate às desigualdades, sobretudo no que diz respeito às mulheres negras, garantindo um acesso mais justo e equitativo aos cuidados em saúde bucal.

## Data Availability

O banco de dados e os códigos de análise da PNS utilizados na pesquisa estão disponíveis em https://www.ibge.gov.br/estatisticas/sociais/saude/9160-pesquisa-nacional-de-saude.html (37), já os aspectos metodológicos, plano amostral e análise de dados estão disponíveis em https://www.scielo.br/j/ress/a/RdbtmCHjJGt8xDW6bV3Y6JB/ (18).
